# Reduced TUBA1A Tubulin Causes Defects in Trafficking and Impaired Adult Motor Behavior

**DOI:** 10.1523/ENEURO.0045-20.2020

**Published:** 2020-04-27

**Authors:** Georgia Buscaglia, Kyle R. Northington, Jeffrey K. Moore, Emily Anne Bates

**Affiliations:** 1 Department of Pediatrics, University of Colorado Anschutz Medical Campus, Aurora CO 80045; 2 Department of Cell and Developmental Biology, University of Colorado Anschutz Medical Campus, Aurora, CO 80045

**Keywords:** ataxia, microtubule network, movement disorder, trafficking, TUBA1A, tubulin

## Abstract

Newly born neurons express high levels of TUBA1A α-tubulin to assemble microtubules for neurite extension and to provide tracks for intracellular transport. In the adult brain, *Tuba1a* expression decreases dramatically. A mouse that harbors a loss-of-function mutation in the gene encoding TUBA1A (*Tuba1a^ND/+^*) allows us to ask whether TUBA1A is important for the function of mature neurons. α-Tubulin levels are about half of wild type in juvenile *Tuba1a^ND/+^* brains, but are close to normal in older animals. In postnatal day (P)0 cultured neurons, reduced TUBA1A allows for assembly of less microtubules in axons resulting in more pausing during organelle trafficking. While *Tuba1a^ND/+^* mouse behavior is indistinguishable from wild-type siblings at weaning, *Tuba1a^ND/+^* mice develop adult-onset ataxia. Neurons important for motor function in *Tuba1a^ND/+^* remain indistinguishable from wild-type with respect to morphology and number and display no evidence of axon degeneration. *Tuba1a^ND/+^* neuromuscular junction (NMJ) synapses are the same size as wild-type before the onset of ataxia, but are reduced in size in older animals. Together, these data indicate that the TUBA1A-rich microtubule tracks that are assembled during development are essential for mature neuron function and maintenance of synapses over time.

## Significance Statement

Defects in axonal trafficking have been reported in models of movement disorders, but it has been unclear whether trafficking defects are coincident or causative. Our results suggest that deficits in TUBA1A α-tubulin can cause trafficking defects that impair synaptic maintenance leading to a movement disorder without axon degeneration or impacting myelination or neuron survival.

## Introduction

Microtubules are the tracks on which proteins, RNA, and organelles are trafficked. Microtubule polymers are composed of αβ-tubulin heterodimers. In humans, nine α-tubulin and 10 β-tubulin isotypes are differentially expressed and could influence properties of specific cellular microtubule networks (Gadadhar et al., 2017). In developing neurons, the most highly expressed α-tubulin is encoded by *Tuba1a* (Lewis et al., 1985; Miller et al., 1987; Gloster et al., 1994, 1999; Zhang et al., 2014). Heterozygous mutations that disrupt human TUBA1A are associated with severe brain malformations, termed tubulinopathies, suggesting that TUBA1A is critical for brain development (Keays et al., 2007; Kumar et al., 2010; Cushion et al., 2013; Aiken et al., 2017). The most common human TUBA1A variants dominantly disrupt microtubule function in neurons (Tian et al., 2010; Aiken et al., 2019b). No deletions, frameshift, or nonsense mutations have been identified in association with TUBA1A tubulinopathy patients (Fallet-Bianco et al., 2008; Cushion et al., 2013; Oegema et al., 2015). We recently proposed TUBA1A mutations that impair brain development dominantly disrupt microtubule function, while heterozygous TUBA1A loss-of-function mutations are tolerable for brain development and are not clinically reported (Aiken et al., 2019a). How TUBA1A contributes to adult neuron function has not been reported.

*TUBA1A* constitutes ∼95% of neuronal α-tubulin mRNA developmentally (Miller et al., 1987). While *Tuba1a* expression is reduced overall in the adult brain, some regions including the hippocampus, cerebellum, basal ganglia, and certain regions of the cerebral cortex retain *TUBA1A* expression in adulthood (Bamji and Miller, 1996). Over time, neuronal microtubule networks acquire post-translational modifications (PTMs) and associations with microtubule-associated proteins (MAPs) that stabilize neuronal microtubules (Brady et al., 1984; Yan et al., 1985; Audebert et al., 1994; Bonnet et al., 2001; Song et al., 2013; Natarajan et al., 2017). TUBA1A proteins may incorporate into microtubules during neurodevelopment and remain within the microtubule network of adult neurons. Indeed, TUBA1A, and other tubulins, have a half-life on the order of weeks to months in rodents (Heo et al., 2018). TUBA1A may be important in the adult neuronal microtubule network. However, mechanistic understanding of TUBA1A function *in vivo* has been limited by the high degree of sequence similarity between α-tubulin isotypes, as isotype-specific α-tubulin antibodies are not available.

To overcome the technical limitations to studying the specific impact of TUBA1A in adult neurons, we use the *Tuba1a^ND^* mutant mouse which harbors an asparagine to aspartic acid substitution at amino acid residue 102 (Gartz Hanson et al., 2016). Homozygous *Tuba1a^ND/ND^*mice are neonatal lethal and have severe brain malformations consistent with homozygous *Tuba1a^null^* and *Tuba1a^quas^* mutant mice (Bittermann et al., 2019) and similar to phenotypes observed in human patients with heterozygous *TUBA1A* mutations (Gartz Hanson et al., 2016; Aiken et al., 2017). The corresponding ND substitution in the primary α-tubulin in yeast decreased α-tubulin protein levels, altered microtubule dynamics, reduced incorporation of the mutant α-tubulin into the lattice, and impaired mitotic division (Gartz Hanson et al., 2016). Together, these data demonstrate that *Tuba1a^ND^*substitution results in less stable microtubules. In contrast to the severe impact of *Tuba1a^ND/ND^*homozygous substitution on neuronal development, heterozygous *Tuba1a^ND/+^*mice survive to adulthood (Gartz Hanson et al., 2016). The *Tuba1a^ND/+^*heterozygous mice can be used to interrogate the requirement for TUBA1A neuronal microtubules within the mature brain.

Here, we show that reduced function of TUBA1A, a tubulin isotype that has historically been characterized only in a developmental context, impacts the ability to maintain synapses over time and ultimately results in an adult-onset movement disorder. Microtubule tracks are not adequately assembled when there is a deficit in TUBA1A tubulin early in development. Insufficient microtubule tracks result in organelle trafficking deficits that are apparent in *Tuba1a^ND/+^*heterozygous neurons early in development. Despite microtubule track abnormalities and resulting changes in trafficking, *Tuba1a^ND/+^*microtubules are sufficient to support survival and morphologically normal neuromuscular junction (NMJ) synapses. However, NMJ synapse morphology and animal behavior deteriorate in an age-related manner in *Tuba1a^ND/+^*animals, without evidence of neuronal cell death or degeneration. Together, these data indicate that both developing and adult neurons require functional *TUBA1A*, and suggest that the “developmental” tubulin *TUBA1A* is required for mature neuronal function.

## Materials and Methods

### Mice

All animal research was performed in accordance with the Institutional Animal Care and Use Committee at the University of Colorado School of Medicine. The *Tuba1a^ND^* mouse model was generated via an ENU-induced mutagenesis forward genetic screen and is described in more detail in Gartz Hanson et al. (2016). All mice used were maintained on a 129S1/C57Bl6 genetic background. Mice were kept on a 12/12 h light/dark cycle with *ad libitum* access to food and water. *Tuba1a^ND^* and wild-type littermate mice were maintained on water supplemented with 0.2 g/l MgSO_4_ to promote *Tuba1a^ND/+^* survival and ability to reproduce. Male and female mice were represented in all studies. All mice were genotyped by PCR amplification of tail DNA followed by Sanger sequencing to differentiate homozygous or heterozygous *Tuba1a^ND/+^*mice from wild type.

### Behavioral phenotyping

Mice were assessed for abnormal motor function between two and 10 months of age. Mice were allowed to acclimate to the testing room for 30 min before behavioral assessment. Gait analysis testing was performed in an enclosed raised chamber with a strip of paper lining the bottom. Fore and hind paws were painted differing colors just before testing, after which mice were placed at the chamber entrance and allowed to run through. Distances between footprints taken from a running gait were analyzed in ImageJ (ImageJ; National Institutes of Health). Rotarod performance was assessed using a Rota-Rod Treadmill (Med Associates, Inc) apparatus with one mouse per lane and a maximum of four mice tested simultaneously. Mice received no prior training on the rotarod task. The rod initially began rotating at 3 RPM and increased until reaching a terminal rotational speed of 30 RPM after 3 min. The time until each mouse fell off or lost its grip such that it could not remain upright on the rod was measured. A cutoff time of 4 min was established for any mice that remained on the rod after peak rotational speed was reached. Mice performed three subsequent trials on the rotarod per testing session, with a 15-min rest period in between trials. Grip strength was tested in mouse forelimbs using a grip strength meter (Stoelting Co). Three subsequent forelimb grip strengths were recorded per testing session.

Mouse hind-paw sensory function was tested between three and six months.

For both Von Frey and Hargreaves analyses, individual mice were contained in 8 × 8 × 17 cm chambers and were allowed to acclimate to the testing chamber for 30 min. Mice were only tested when resting on all four paws in a non-grooming awake state. Von Frey analysis of mechanical stimulus sensation was performed in left and right hind-paws using Touch Test Sensory Probes (Stoelting Co). Von Frey performance score was analyzed using the SUDO method established in Bonin et al. (2014). Hargreaves analysis of thermal pain sensation was assessed using a paw thermal stimulator instrument (University of California, San Diego). Time until withdrawal from thermal stimuli was recorded for each animal in both left and right hind-paws. Three subsequent trials were performed for each hind-paw per testing session.

### Histology

Mice were anesthetized and transcardially perfused with 0.1 M NaCl and 4% paraformaldehyde (PFA) for histology. Tissues of interest were dissected and postfixed in 4% PFA. Serial tissue sectioning was performed on a CM1520 cryostat (Leica) and 30-μm serial cryosections were obtained for all histology experiments. For all histologic analyses, three regions were chosen for quantification which spanned the anterior-posterior axis, and the same regions were assessed for each animal. For Nissl staining, sections from the lumbar spinal cord were stained using 0.1% cresyl violet. For immunofluorescence studies, PFA-fixed tissues were blocked in PBS containing 5% bovine serum albumin (BSA) with 0.3% Triton X-100. Primary and secondary antibodies were diluted in PBS containing 1% BSA with 0.1% Triton X-100. For α-bungarotoxin labeling of NMJ synapses, PFA-fixed extensor digitorum longus (EDL), flexor digitorum brevis, and soleus muscles were dissected from the hindlimbs of postnatal day (P)30 and adult mice. Muscle fibers were teased apart using forceps and mounted onto positively charged glass microscope slides. Muscle fibers were blocked in a blocking buffer containing 3% BSA, 5% goat serum, and 0.5% Triton X-100 and then stained with primary antibodies diluted in blocking buffer at 4°C overnight. Secondary antibodies and rhodamine-conjugated α-bungarotoxin (a generous gift from Dr. John Caldwell) were added for two hours at room temperature. Primary antibodies were as follows: mouse anti-calbindin D-28k (Swant AgCB10abs; 1:250), rabbit anti-ER81 (BioLegend 840401; 1:5000), mouse anti-SMI32 (BioLegend 801702; 1:5000), rabbit anti-synaptophysin (Invitrogen MA5-14 532; 1:200). Fluorescently conjugated secondary antibodies were Life Technologies all used at 1:500. For NMJ analysis, Z-stack images were obtained at 63× magnification with a slice size of 0.5 μm with 10 total slices covering 4.5-μm total z-distance.

### Electron microscopy

Mice used for electron microscopy were perfused with 0.1 M NaCl and 2.5% glutaraldehyde 4% PFA, after which the spinal column was dissected and postfixed in 2.5% glutaraldehyde 4% PFA overnight at 4°C. Following postfixation, spinal cords were dissected from the spinal column, and a 2-mm region of the cervical spinal cord between C2 and C4 was dissected and sent for further processing and imaging by the CU School of Medicine Electron Microscopy Core facility. Myelin thickness, axon density, and axon diameter were measured from electron micrographs in the lumbar spinal cord. G-ratio was calculated using the formula G = r_i_/R_i_ where r_i_ = radius of axon and R_i_ = radius of axon and myelin. At least three sections per animal were analyzed, measuring 100 axons per animal. The same total area was quantified between wild-type and *Tuba1a^ND/+^* mice.

### Cell culture and transfection

Dissociated neurons were cultured from post-natal day 0 (P0) to P2 mouse cortices. Brains were removed and placed into HBSS (Life Technologies) supplemented with 1 M HEPES (Life Technologies) and 1 mM kynurenic acid (Tocris Bioscience). Meninges were removed and cortices were dissected and cut into ∼1-mm pieces. Cortical pieces were triturated to a single-cell suspension using glass Pasteur pipettes. Cortical neurons were plated onto 35-mm Poly-D-Lysine coated glass-bottom culture dishes at a density of 350,000 cells/35 mm. Neurons were maintained in a 37°C humidified incubator with 5% CO_2_ in phenol-free Neurobasal-A medium (Life Technologies) supplemented with B-27 (Thermo), penn/strep (Thermo), GlutaMax (Thermo), 5 ng/ml β-FGF (Invitrogen), and sodium pyruvate (Thermo). For GFP-MACF43 puncta analysis, neurons were nucleofected with 2 μg of plasmid DNA using a mouse neuron nucleofector kit (Lonza) and Nucleofector 2b Device (Lonza), then plated normally.

### GFP-MACF43 and organelle transport assays

Primary cortical neurons nucleofected with GFP-MACF43 plasmid DNA were imaged at day in vitro (DIV) 1 to estimate the number of microtubule (+)-ends per μm of axon. Cells were placed in a 37°C imaging chamber and imaged using a 40× oil objective on a Zeiss 780 confocal microscope using ZEN imaging software (Zeiss). GFP-MACF43 images were acquired every 2 s for a total duration of 2 min. Multiple 2-min videos were acquired for each neuron imaged, and data were used to generate a cell average, which was reported. Lysosomal and mitochondrial trafficking was assessed in cortical neurons at DIV7. Cortical neurons were incubated in either LysoTracker Red DND-99 or MitoTracker Red FM (Invitrogen) dye for 20 min at 37°C, after which normal culture media was replaced and cells were transferred to a 37°C imaging chamber and imaged on either a Zeiss 780 confocal (MitoTracker) or a Nikon Ti-E microscope equipped with a 1.3 NA 40× CFI60 Plan Fluor objective, piezo electric stage (Physik Instrumente), spinning disk confocal scanner unit (CSU10; Yokogawa), 488 nm and 561 nm lasers (Agilent Technologies), and an EMCCD camera (iXon Ultra 897; Andor Technology) using NIS Elements software (Nikon). Cells were imaged every 1 s or every 2 s for lysosomes and mitochondria, respectively.

### Western blotting

Protein was isolated from brains of P0–P2 and adult mice by dounce homogenization and ultra-centrifugation, using a modified version of the protocol described by Vallee (1982). Protein concentrations were assessed using a BCA assay (Thermo), and relative concentration was determined using a Synergy H1 microplate reader (BioTek Instruments). For tubulin Western blotting, 0.25 μg of brain lysate was loaded per lane, for all other blots 10 μg of tissue lysate was used. Protein was run on 4–20% gradient acrylamide gels (Bio-Rad Laboratories) at 150 mV for 1 h. Proteins were transferred to PVDF blotting membranes (Bio-Rad) in transfer buffer containing 25 mM Tris-base, 192 mM glycine, and 15% methanol for 1 h at 75 V at 4°C. Gels were dyed with Coomassie Blue (Bio-Rad) post-transfer to assess transfer efficiency for each blot. Membranes were blocked in Tris-buffered saline containing 0.1% Tween 20 (TBST) with 5% BSA for 1 h and incubated in primary antibody overnight at 4°C. Primary antibodies were diluted in TBST containing 1% BSA at 4°C overnight. Blots were incubated in secondary antibodies diluted in TBST containing 0.5% BSA with streptavidin-HRP (Bio-Rad, 1:10,000) for 1 h at room temperature. Primary antibodies for Western blotting were as follows: mouse anti-DM1A (Sigma-Aldrich T6199; 1:10,000), rabbit anti-GAPDH (Cell Signaling 14C10; 1:1000), mouse anti-acetylated tubulin (Sigma-Aldrich T7451; 1:1000), mouse anti-tyrosinated tubulin (Sigma T9028; 1:5000), rabbit anti-detyrosinated tubulin (Abcam ab48389; 1:1000), mouse anti-polyglutamylated tubulin (GT335, Adipogen AG-20B-0020; 1:2000), and mouse anti-SMI32 (BioLegend 801702; 1:500). Secondary antibodies used were HRP conjugated and diluted 1:5000 (Santa Cruz Biotechnology). Blots were developed in ECL solution (Bio-Rad) and imaged using a ChemiDoc MP imager (Bio-Rad).

### RNA isolation + RTPCR

RNA was isolated from flash-frozen mouse brains dissected from P0 or adult mice using an SV Total RNA Isolation system (Promega). RNA concentration and purity were determined using a spectrophotometer, then cDNA was synthesized using the RT2 First Strand kit (QIAGEN). RT-PCR reactions were prepared with SYBR Green RT-PCR Master mix (Thermo) and run with a CFX Connect Real-Time System (Bio-Rad). Samples were run in triplicate, results were analyzed in Excel. All qPCR data presented in this manuscript was normalized to expression of the housekeeping gene Cyclin A. *Tuba1a^ND/+^* relative mRNA quantity was normalized to wild type in cases where mRNA expression was compared by genotype. In cases where tubulin genes were compared with one another, we calculated relative quantity of mRNA (RQ) using the equation RQ = E ^⋀^-ΔCT, where E is the primer efficiency, calculated from running a dilution curve of each primer set. For all qRT-PCR experiments, three biological replicates were used per genotype. Primers were as follows: TUBA1A, F: ATTATGAGGAGGTTGGTGTG and R: TGTTGGAACACAATAAACATC; TUBA1B, F: CTCATTGCGTTACTTACCTC and R: TCACGCATGATAGCAACG; TUBA1C, F: GGTATATAAGCCCTGTCCTG and R: CTCACGCATATTTAGTCCTTG; and TUBA4A, F: ACTGTAATCGATGAGATCCG and R: AATACTAGGAAGCCCTGAAG.

### Experimental design and statistical analyses

All experiments used experimental design statistics for random data. Band volume of all Western blottings was analyzed using Image Lab software (Bio-Rad). Organelle transport and GFP-MACF43 comet analysis, kymograph generation, and assessment of EM images was performed using publicly available plugins for FIJI software (National Institutes of Health). Maximum intensity projection images from en face NMJ synapses were analyzed for synaptic area, presynaptic and postsynaptic marker intensities using custom MATLAB software (MathWorks 2018). Cell counting was performed either using FIJI or MATLAB software. Statistical analyses were performed, and graphs were created using Prism version 8.0 (GraphPad). Most graphs display all data points to accurately represent the variability in each dataset, except in cases where such presentation obscured the conclusion. For all statistical analyses, means were considered to be significantly different if *p* < 0.05. Statistical analyses used in each experiment are indicated in their respective figure legends. For all graphs mean ± SEM was reported unless otherwise noted. Normality of each dataset was assessed using a Shapiro–Wilk test. In datasets with two groups, parametric data were analyzed using a Student’s *t* test, while non-parametric data were assessed by Mann–Whitney *U* analysis of medians. Multiple groups were compared by one-way or two-way ANOVA and analyzed *post hoc* by either a Bonferroni or Kruskal–Wallis test for parametric and non-parametric data, respectively. Detailed information on how each in-text statistical figure was reached can be found in Table 1.

**Table 1:** Statistical table

	Data structure	Type of test	Power
1	Normal distribution	Two-way ANOVA	0.4478 to 1.369
2	Normal distribution	Unpaired *t* test	–0.03822 to 0.02562
3	Normal distribution	Two-way ANOVA	–0.6247 to 0.1850
4	Normal distribution	Unpaired *t* test	–2.839 to 1.889
5	Normal distribution	Unpaired *t* test	–0.5567 to 0.9367
6	Normal distribution	Unpaired *t* test	–0.3997 to 0.3219
7	Normal distribution	Unpaired *t* test	–0.5390 to 1.306
8	Normal distribution	Two-way ANOVA	–1.079 to 1.932
9	Normal distribution	Two-way ANOVA	–1.272 to 1.272
10	Normal distribution	Two-way ANOVA	–0.5164 to 0.2583
11	Normal distribution	Two-way ANOVA	–0.6096 to 0.6096
12	Normal distribution	Unpaired *t* test	–2.814 to –1.238
13	Normal distribution	Unpaired *t* test	1.294 to 26.44
14	Log-normal distribution	Welch's *t* test	–0.08284 to 0.02308
15	Log-normal distribution	Mann–Whitney *U*	–0.5000 to 1.500 (approximate)
16	Normal distribution	Unpaired *t* test	–12.53 to –2.627
17	Normal distribution	Unpaired *t* test	–14.22 to 5.521
18	Log-normal distribution	Welch's *t* test	–0.06821 to 0.03392
19	Log-normal distribution	Mann–Whitney *U*	0.5000 to 4.834 (approximate)
20	Normal distribution	Unpaired *t* test	–10.51 to –2.180
21	Normal distribution	Two-way ANOVA	–0.6070 to –0.2015
22	Normal distribution	Two-way ANOVA	51.50 to 237.4
23	Normal distribution	Linear regression	–18.51 to 65.11 (WT); –53.22 to 83.22 (Tuba1aND)
24	Normal distribution	Unpaired *t* test	–6.520 to 1.782
25	Normal distribution	Unpaired *t* test	–0.07948 to 0.3509
26	Normal distribution	Unpaired *t* test	0.1362 to 3.864
27	Normal distribution	Unpaired *t* test	–1.274 to 5.889
28	Normal distribution	Two-way ANOVA	–111.3 to 17.97 (3 months); –83.08 to 46.19 (10 months)
29	Normal distribution	Two-way ANOVA	–9.574 to 6.240 (3 months); –12.80 to 3.018 (10 months)
30	Normal distribution	Two-way ANOVA	–360.1 to 129.1 (3 months); –277.1 to 212.1 (10 months)
31	Normal distribution	Unpaired *t* test	–0.03303 to 0.05303
32	Normal distribution	Unpaired *t* test	–0.1935 to 0.1596
33	Normal distribution	Unpaired *t* test	–0.5387 to 0.7019
34	Normal distribution	Unpaired *t* test	–608.3 to 741.8
35	Normal distribution	Unpaired *t* test	–0.8969 to 1.449 (NFT-M); –2.403 to 3.450 (NFT-H)
36	Normal distribution	Unpaired *t* test	–113.7 to –37.30
37	Normal distribution	Unpaired *t* test	–0.04520 to 0.08451 (pre); –0.1550 to 0.1791(post)
38	Normal distribution	Unpaired *t* test	–0.1173 to 0.1639
39	Normal distribution	Unpaired *t* test	–116.1 to –56.69
40	Normal distribution	Unpaired *t* test	0.1642 to 0.3719
41	Normal distribution	Unpaired *t* test	0.1905 to 0.4305
42	Normal distribution	Unpaired *t* test	–0.3050 to –0.01338

## Results

### *Tuba1a* is a major component of adult α-tubulin mRNA

**Figure 1.**
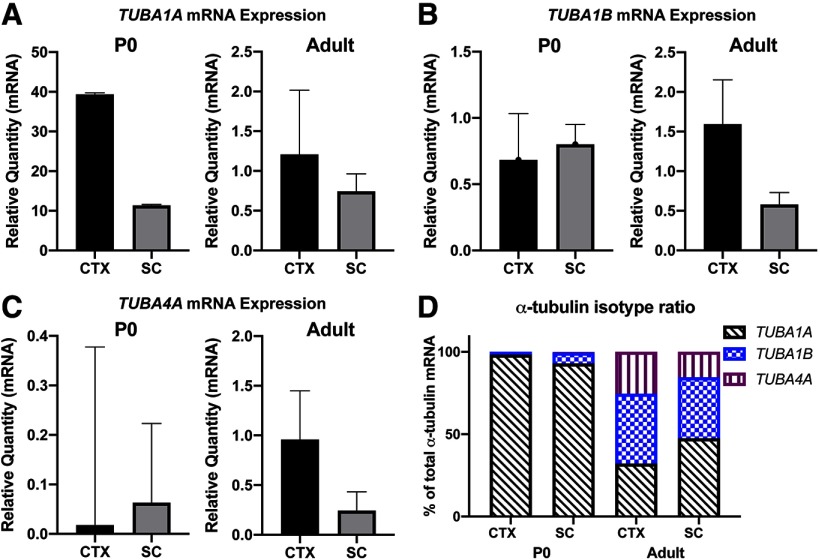
Changes in α-tubulin isotype expression in cortex (CTX) and spinal cord (SC) between post-natal day 0 (P0) and adult. ***A***, Bar graphs representing relative quantity of *TUBA1A* mRNA in P0 (left) and adult (right) mouse cortex and spinal cord. ***B***, Bar graph representing relative quantity of *TUBA1B* mRNA in cortex and spinal cord of P0 (left) and adult (right) mice. ***C***, Bar graph representing relative quantity of *TUBA4A* mRNA in cortex and spinal cord of P0 (left) and adult (right) mice. ***D***, Bar graphs representing the α-tubulin isotype mRNA expression data from ***A–C*** as a percentage of the total α-tubulin isotype composition. Three animals per genotype were analyzed for each time point, with three technical replicates per animal. Data are plotted as relative mRNA quantity normalized to housekeeping gene Cyclin A. Bars represent mean ± SEM.

During embryonic development, *Tuba1a* constitutes ∼95% of all α-tubulin mRNA in the mouse brain (Miller et al., 1987). By comparison, postnatal *Tuba1a* expression is dramatically decreased, however certain brain regions including the neocortex and hippocampus retain *Tuba1a* expression into adulthood (Bamji and Miller, 1996). Many studies have focused on the role of *Tuba1a* during brain development, but it is unclear how much *Tuba1a* contributes to the α-tubulin pool in adult neurons of the brain and spinal cord. Although *TUBA1A* is ubiquitously expressed in postmitotic neurons, the relative abundance of different α-tubulin isotypes in specific neuronal subtypes over time has not been well characterized. To understand how the neuronal α-tubulin isotype blend changes over time, we assessed mRNA for *Tuba1a*, *Tuba1b*, *Tuba1c*, and *Tuba4a* in the cortex and spinal cord of post-natal day 0 (P0) and adult wild-type mice (Fig. 1*A–C*; *N* = 3). Unfortunately, levels of *Tuba1c* mRNA were too low to be reliably detected in our assays, thus we have omitted those data. Although the relative quantity of mRNA for each isotype differed between the cortex and spinal cord, we found that the ratio of α-tubulin isotypes was comparable between spinal cord and cortex, in both newborn and adult animals (Fig. 1*D*; *N* = 3). Intriguingly, although *Tuba1a* expression is downregulated between development and adulthood, we found that in wild-type adult mice, *Tuba1a* comprises 32% of all cortical and 47% of all spinal cord α-tubulin mRNA (Fig. 1*D*). These data demonstrate that although *Tuba1a* mRNA abundance decreases postnatally, *Tuba1a* is the most abundant α-tubulin isotype in adult spinal cord and the second-most abundant α-tubulin in adult cortex. Because *Tuba1a* constitutes a large percentage of the total α-tubulin in both cortical and spinal neurons over time, we predict that *Tuba1a* plays an important role in the adult neuronal microtubule network.

### *Tuba1a^ND^* is a loss-of-function mutation in mice

**Figure 2.**
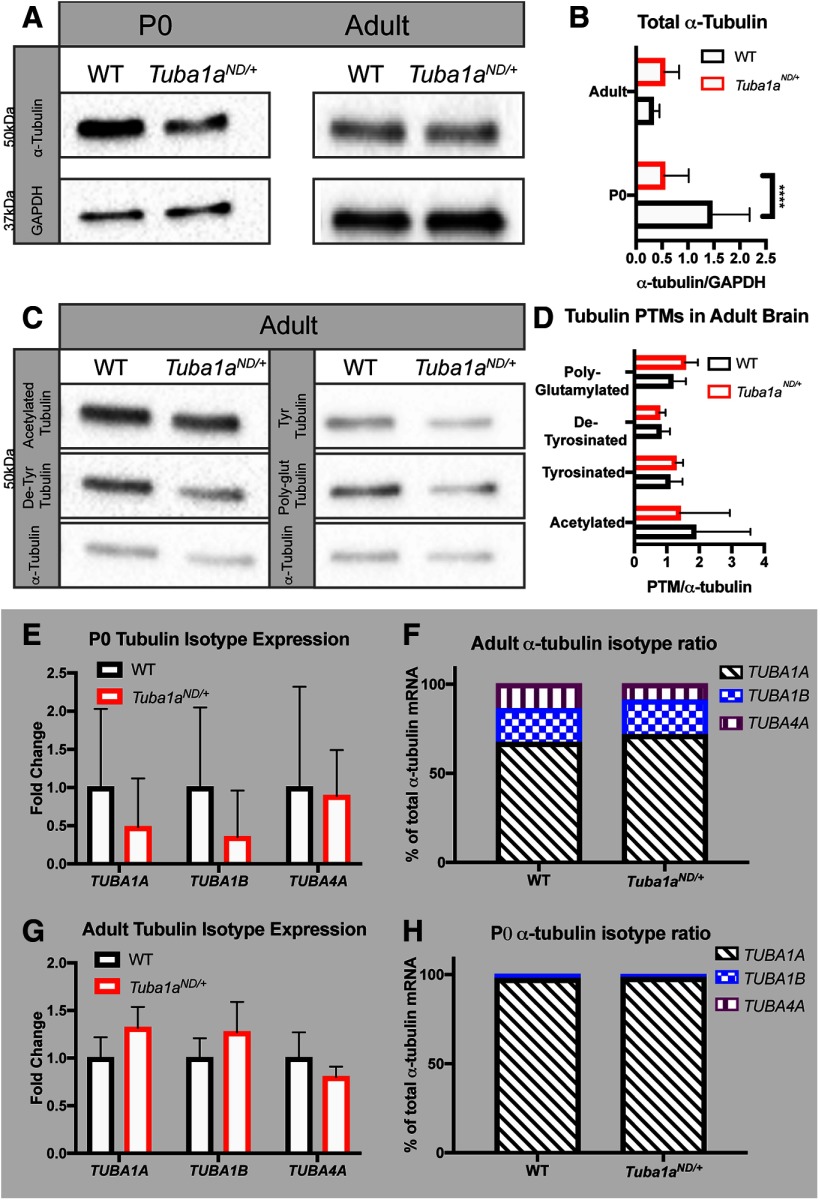
α-Tubulin protein is decreased in P0 *Tuba1a^ND/+^* brains. ***A***, Western blottings representing α-tubulin abundance in P0 (left) and adult (right) *Tuba1a^ND/+^* and wild-type (WT) mouse brains. ***B***, Bar graph quantifying α-tubulin protein in P0 and adult brain tissue lysates relative to GAPDH (*N* = 3 mice, *p* < 0.0001 by two-way ANOVA). Quantifications are representative of at least three separate experiments. ***C***, Western blottings showing the abundance of α-tubulin PTMs acetylation (top left), tyrosination (top right), detyrosination (center left), and polyglutamylation (center right), and total α-tubulin (bottom) in adult wild-type and *Tuba1a^ND/+^*whole-brain lysates. ***D***, Bar graph quantification of Western blotting data shown in ***C***. PTM band volume was normalized to α-tubulin (*p* > 0.05 for all by *t* test). ***E***, ***F***, mRNA expression of brain α-tubulin isotypes in *Tuba1a^ND/+^*and wild-type mice at P0. Data are plotted as fold change in *Tuba1a^ND/+^*relative to wild type (***E***) or as a percentage of the total α-tubulin isotype composition (***F***; *p* > 0.05 for all by two-way ANOVA). ***G***, ***H***, Bar graph representing mRNA expression of brain α-tubulin isotypes in adult *Tuba1a^ND/+^*and wild-type mice. Data are plotted as fold change in *Tuba1a^ND/+^*relative to wild type (***G***) or as a percentage of the total α-tubulin isotype composition (***H***; *p* > 0.05 for all by two-way ANOVA). All Western blotting data represents three replicate blots with lysates from at least three animals per genotype. Bars represent mean ± SEM. ****p < 0.0001.

While it is known that Tuba1a contributes significantly to development, it has been difficult to determine how this isotype contributes to adult neuronal function due to the lack of available tools to study *Tuba1a* protein. Previous data in yeast (Gartz Hanson et al., 2016) suggested that heterozygous *Tuba1a^ND/+^*mice may have reduced *Tuba1a* function. To determine whether the *Tuba1a^ND^* substitution affects levels of α-tubulin in mice, we isolated protein from wild-type and *Tuba1a^ND/+^*brains at P0–P2. We did not include homozygous *Tuba1a^ND/ND^*mouse brain lysates due to their perinatal lethality. Quantitative Western blottings revealed an approximate 50% decrease in α-tubulin protein in heterozygous *Tuba1a^ND/+^* mouse brains compared with wild-type at birth (Fig. 2*A*,*B*; *N* = 4, *p* < 0.0001) consistent with the molecular impact of the ND substitution in yeast reported in Hanson et al., (2016). This reduction in α-tubulin protein was not due to a change in brain size, as weights between wild-type and *Tuba1a^ND/+^*brains were comparable (Extended Data Fig. 2-1; *N* = 10, *p* = 0.68). Adult *Tuba1a^ND/+^* α-tubulin protein levels were comparable to wild type (Fig. 2*A*,*B*; *N* = 6, *p* = 0.38). The observed reduction in developmental α-tubulin indicates that the availability of α-tubulin protein is decreased in *Tuba1a^ND/+^*neurons during a critical period for neuronal microtubule network establishment. As total α-tubulin protein levels normalized in the brains of adult *Tuba1a^ND/+^*mice, we assessed brain tubulin PTMs because PTMs influence binding interactions with MAPs and overall microtubule function. Western blottings of whole-brain lysates from adult wild-type and *Tuba1a^ND/+^*mice revealed no genotypic difference in the abundance of acetylated (*N* = 5; *p* = 0.66), tyrosinated (*N* = 3; *p* = 0.52), detyrosinated (*N* = 5; *p* = 0.81), or polyglutamylated (*N* = 3; *p* = 0.31) α-tubulin relative to the total α-tubulin (Fig. 2*C*,*D*). Therefore, we conclude that *Tuba1a^ND^*substitution reduces the availability of α-tubulin protein developmentally, but does not impact the PTM landscape of adult brain microtubules.

10.1523/ENEURO.0045-20.2020.f2-1 Extended Data Figure 2-1 ***A***, *Tuba1a^ND/+^*does not alter brain weight at birth. Scatter plot of brain weight for *Tuba1a^ND/+^*and wild-type mice at P0–P2 (*N* = 10 mice, *p* = 0.68 by *t* test). Weights were recorded from frozen, dissected brains. Download Figure 2-1, TIF file.

Mice have nine α-tubulin genes and mammalian neurons express at least four distinct α-tubulin genes (Miller et al., 1987; Bamji and Miller, 1996; Gloster et al., 1999; Zhang et al., 2014). Mutations that disrupt the major isoform, *Tuba1a*, could potentially induce compensatory changes to the expression of other α-tubulin mRNAs. We found that mRNA expression of *Tuba1a* and other brain α-tubulin isotypes was not significantly changed in heterozygous *Tuba1a^ND/+^*P0 brain lysates (Fig. 2*E*; *p* > 0.9 for all comparisons by two-way ANOVA). Additionally, the relative ratio of each α-tubulin isotype mRNA was unchanged in *Tuba1a^ND/+^* P0 brain lysates, with *Tuba1a* constituting ∼98% of all α-tubulin mRNA in both wild-type and *Tuba1a^ND/+^*brains (Fig. 2*F*; *N* = 3, *p* = 0.92). We conclude from these results that alternative α-tubulin isotypes are not upregulated to compensate for the loss of TUBA1A protein in the developing brain. We examined the abundance of α-tubulin isotype mRNAs in the cortex of adult wild-type and *Tuba1a^ND/+^*mice and found that *Tuba1a* remains one of the predominant α-tubulins for both genotypes (Fig. 2*G*; *N* = 3; *p* > 0.7 for all by two-way ANOVA). The modest, but statistically insignificant increase in *Tuba1a* mRNA in the adult *Tuba1a^ND/+^*cortex slightly shifted the ratio of α-tubulin isotype mRNAs in the adult cortex, with an increased percentage of Tuba1a α-tubulin mRNA compared with the other isotypes (Fig. 2*H*; *p* < 0.0001 in *Tuba1a^ND/+^*cortex compared with wild-type by two-way ANOVA). Together, these data demonstrate that *Tuba1a^ND/+^*transiently depletes α-tubulin protein during a critical developmental window, without inducing compensatory upregulation of other α-tubulin isotypes.

### Reduced TUBA1A function results in fewer microtubule tracks in neurites

**Figure 3.**
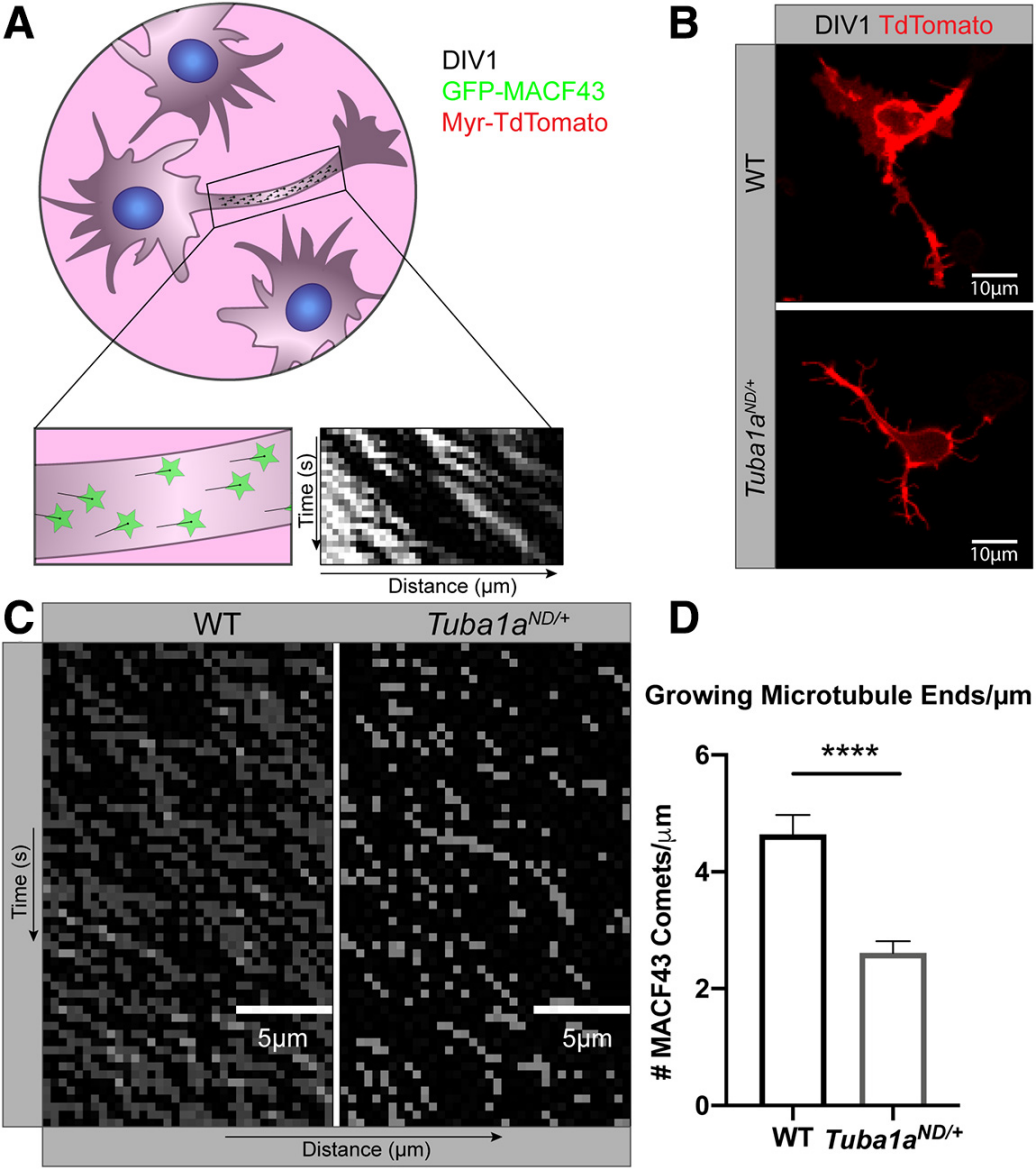
Reduced TUBA1A function results in fewer microtubule tracks in neurites. ***A***, Schematic of experimental design, illustrating microtubule plus-end tracker, GFP-MACF43, puncta analysis and kymograph generation. ***B***, Representative images of day in vitro (DIV) 1 wild-type (WT) and *Tuba1a^ND/+^*neurons, visualized using a membrane-bound Myr-TdTomato. Scale bars: 10 μm. ***C***, Representative kymograph plots generated from GFP-MACF43 images in wild-type (left) and *Tuba1a^ND/+^*(right) neurons. Scale bars: 5 μm. ***D***, Bar graph representing the number of GFP-MACF43 puncta per μm of neurite in DIV1 wild-type and *Tuba1a^ND/+^*cortical neurons. Bars illustrate mean density of GFP-MACF43 puncta per kymograph and error bars indicate SEM (*n* = 24 neurons, *p* < 0.0001). Statistical differences between groups were assessed by *t* test. ****p < 0.0001.

To determine how reduced TUBA1A impacts the development of a neuronal microtubule network, we used primary neurons from wild-type and *Tuba1a^ND/+^*P0–P2 cortices to examine microtubule organization *in vitro*. Wild-type and *Tuba1a^ND/+^* primary cortical neurons were nucleofected with membrane-bound Myr-TdTomato and GFP-MACF43, which binds to polymerizing microtubule (+)-end-binding proteins (EBs) without affecting microtubule function (Yau et al., 2016). Kymograph plots generated from GFP-MACF43 videos allowed us to interrogate the number of growing microtubule (+)-ends per micrometer of neurite at day in vitro (DIV) 1 (Fig. 3*A–C*). We found that *Tuba1a^ND/+^*neurites have approximately half the number of GFP-MACF43 puncta per micrometer of neurite as wild-type neurites [2.610 ± 0.20 growing microtubule ends in *Tuba1a^ND/+^*vs 4.636 ± 0.34 in wild type (Fig. 3*D*); *n* = 24 neurites; *p* < 0.0001]. These data suggest the approximate 50% reduction in α-tubulin protein in observed in developmental *Tuba1a^ND/+^*mice causes functional loss of microtubule track density within developing *Tuba1a^ND/+^*neurons.

### TUBA1A is required for functional intracellular transport

One critical function of neuronal microtubules is to facilitate intracellular trafficking of proteins, mRNAs, and organelles between the cell body and distal neurites. Thus, we explored the possibility that reduced TUBA1A function and a reduced density of microtubule tracks impairs microtubule-based intracellular transport. To evaluate how loss of TUBA1A impacts the dynamics of microtubule-based transport, we examined transport of lysosomes in cultured primary neurons from P0–P2 wild-type and *Tuba1a^ND/+^* cortices.

**Figure 4.**
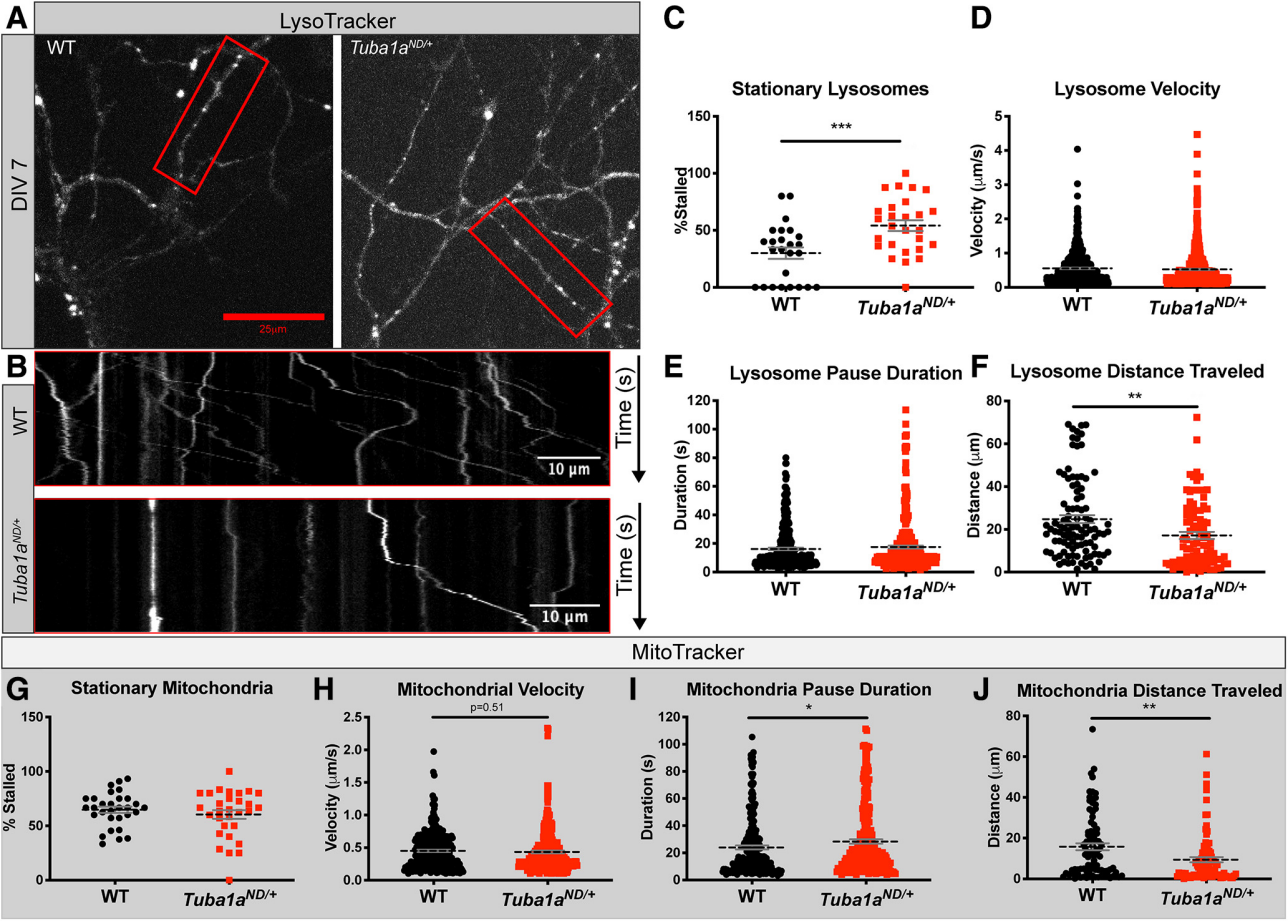
*Tuba1a^ND/+^*neurons have deficits in organelle transport caused by increased stationary cargoes. ***A***, Still images from time-lapse microscopy of DIV3 cortical neurons labeled with LysoTracker dye to mark lysosomes in wild-type (WT) and *Tuba1a^ND/+^*neurons. ***B***, Representative kymograph plots of lysosome movement over time within select neurites for wild type (top) and *Tuba1a^ND/+^*(bottom). ***C***, Scatter plot representing the percent of stationary lysosomes per kymograph in wild-type and *Tuba1a^ND/+^*neurons. Data points represent individual neurons (*N* = 3 mice, *n* = 25 neurons, *p* = 0.001). ***D***, Scatter plot representing lysosome velocities in μm/s for wild-type and *Tuba1a^ND/+^*neurons. Data points represent individual lysosomes (*n* = 635 lysosomes, *p* = 0.27). ***E***, Scatter plot representing pause duration (s), for moving lysosomes in wild-type and *Tuba1a^ND/+^*neurons. Data points represent individual pause events (*n* = 338 events, *p* = 0.56). ***F***, Scatter plot representing total distance traveled (μm) by moving lysosomes in wild-type and *Tuba1a^ND/+^*neurons. Data points represent individual lysosomes (*n* = 87, *p* = 0.003). ***G***, Scatter plot representing the percentage of stationary mitochondria in wild-type and *Tuba1a^ND/+^*neurons. Data points represent individual neurons (*N* = 3 mice, *n* = 30 neurons, *p* = 0.34 by). ***H***, Scatter plot the velocity of short mitochondrial movements in wild-type and *Tuba1a^ND/+^*neurons. Data points represent individual mitochondria (*N* = 3 mice, *n* = 301 mitochondria, *p* = 0.45). ***I***, Scatter plot representing pause duration (s), for moving mitochondria in wild-type and *Tuba1a^ND/+^*neurons. Data points represent individual pause events (*n* = 417 events, *p* = 0.79). ***J***, Scatter plot representing total distance traveled (μm) by moving mitochondria in wild-type and *Tuba1a^ND/+^*neurons. Data points represent individual mitochondria (*n* = 86, *p* = 0.01). Cortical neurons from three animals per genotype were analyzed. Lysosomes were imaged once every second for 3 min, mitochondria were imaged once every 2 s for 2 min. Statistical differences between groups were assessed by Student’s *t* test; **p < 0.01; ***p < 0.001. In all plots, line designates mean with error bars indicating SEM. *p < 0.05; **p < 0.01; ***p < 0.001.

Lysosomes facilitate enzymatic degradation of intracellular components and are highly motile organelles that are transported bidirectionally on microtubules through all compartments of the neuron, making them an ideal organelle in which we could examine microtubule-based transport efficiency. Time-lapse images of lysosome movement in wild-type or *Tuba1a^ND/+^*cortical neurons were used to generate kymograph plots of lysosome movement over time within single wild-type and *Tuba1a^ND/+^* axons and dendrites (Fig. 4*A*,*B*), from which we could measure velocity, dynamics of movement, and total distance traveled. From these analyses we found that during the 3-min imaging period, ∼70% of wild-type lysosomes were moving, while the remainder were stationary for the duration of the video (Fig. 4*C*; *N* = 3 mice, *n* = 25 neurons). A significantly higher number of *Tuba1a^ND/+^*lysosomes were stationary compared with wild type, with only 46% of *Tuba1a^ND/+^*lysosomes moving during the 3-min window (Fig. 4*C*; *p* = 0.001). Of those lysosomes that were moving, the velocity of each movement was unchanged between wild-type and *Tuba1a^ND/+^*neurons, with average velocity of 0.56 ± 0.02 and 0.53 ± 0.02 μm/s for wild-type and *Tuba1a^ND/+^*neurons, respectively (Fig. 4*D*; *n* = 635 lysosomes, *p* = 0.27). During active transport, cargoes exhibit phases of movement and pausing, thus we wanted to evaluate the dynamics of lysosome movement by measuring pause duration for moving lysosomes. There was no difference in pause duration of moving lysosomes in *Tuba1a^ND/+^*neurons, which paused on average 17.51 ± 1.0 s, compared with a 16.19 ± 0.81-s pause duration in wild-type neurons (Fig. 4*E*; *n* = 337 events, *p* = 0.56). Finally, we observed a significant decrease in the total distance traveled by lysosomes over the 3-min imaging window, from 24.77 ± 1.84 μm in wild-type neurons to 17.19 ± 1.66 μm in *Tuba1a^ND/+^*neurons (Fig. 4*F*; *n* = 100 neurons, *p* = 0.003). These data demonstrate that *Tuba1a^ND/+^*substitution causes a deficit in neuronal intracellular transport by which *Tuba1a^ND/+^*lysosomes do not travel as far as wild type within the same time window.

To further understand the impact of reduced TUBA1A function on neuronal microtubule-based transport, we examined mitochondrial trafficking in cultured primary *Tuba1a^ND/+^*neurons compared with wild type. Transport of mitochondria and lysosomes is conducted by a different set of kinesins (Hurd and Saxton, 1996; Tanaka et al., 1998; Hendricks et al., 2010; Maday et al., 2014). Therefore, examining transport of multiple cargoes provides mechanistic insight into the intracellular trafficking disruption in *Tuba1a^ND/+^*neurons. The analyses described above were repeated in DIV7 wild-type and *Tuba1a^ND/+^*cortical neurons that had mitochondria labeled (Extended Data Fig. 4-1). Over the course of a 2-min imaging period, we observed that the number of moving mitochondria was similar between wild-type (36%) and *Tuba1a^ND/+^*(40%) neurons, and the remainder were stationary for the duration of the video (Fig. 4*G*; *N* = 3 mice, *n* = 30 neurons, *p* = 0.38). Importantly, the relative number of stationary mitochondria was more than double the number of stationary lysosomes in wild-type neurons (64.78 ± 2.9% and 30.01 ± 5.0%, respectively; Fig. 4*C*,*G*). Similar to what was observed for lysosomal trafficking, we found that the velocity of moving mitochondria was unchanged between wild-type (0.45 ± 0.02 μm/s) and *Tuba1a^ND/+^*(0.43 ± 0.02 μm/s) neurons (Fig. 4*H*; *n* = 301 events, *p* = 0.51). These mitochondrial movements were interrupted by differential periods of pause, with a significant increase in *Tuba1a^ND/+^*mitochondrial pausing compared with wild type, with average pause durations of 28.36 ± 1.6 and 24 ± 1.4 s, respectively (Fig. 4*I*; *n* = 251 events, *p* = 0.02). The total distance traveled by motile mitochondria during the 2-min imaging window was found to be significantly decreased in *Tuba1a^ND/+^*neurons, with *Tuba1a^ND/+^*mitochondria traveling 9.46 ± 1.3 μm on average compared with 15.8 ± 1.7 μm in wild-type neurons (Fig. 4*J*; *n* = 301 events, *p* = 0.01). Combined, these analyses indicate that a TUBA1A deficit impairs transport of multiple organelle types in cortical neurons, resulting in a significant reduction in the total distance of organelle movement.

10.1523/ENEURO.0045-20.2020.f4-1 Extended Data Figure 4-1 *Tuba1a^ND/+^*impairs mitochondrial transport by increasing pause duration. ***A***, Still images from time-lapse microscopy of DIV3 cortical neurons labeled with MitoTracker dye to mark mitochondria in wild-type and *Tuba1a^ND/+^* neurons. ***B***, Insets show representative kymograph plots of mitochondrial movement over time within select neurites for wild type (top) and *Tuba1a^ND/+^*(bottom). Download Figure 4-1, TIF file.

### Diminished TUBA1A causes late-onset behavioral deficits

**Figure 5.**
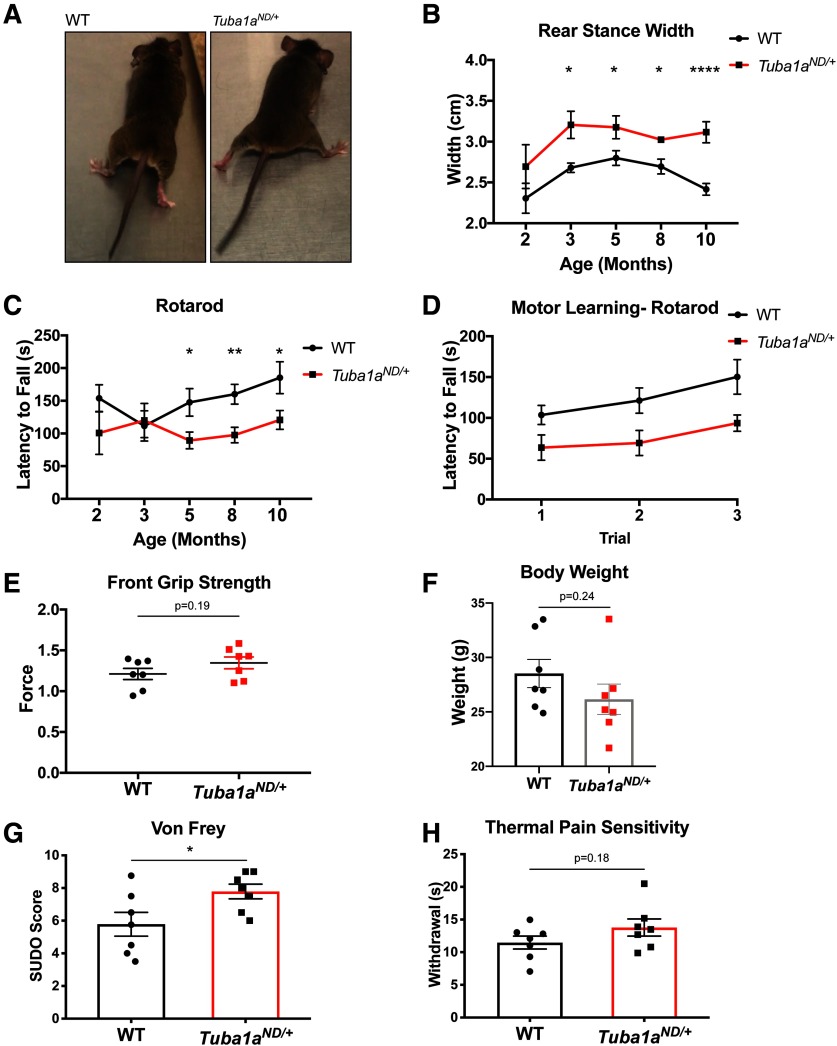
*Tuba1a^ND/+^*mice exhibit adult-onset motor and sensory behavioral deficits. ***A***, Images depicting gait abnormalities in *Tuba1a^ND/+^*adult mice compared with wild-type (WT). Still images were taken from video of *Tuba1a^ND/+^*and wild-type mice mid-stride. ***B***, Line graph quantifying changes in rear stance width at two months (*p* = 0.61), three months (*p* = 0.02), five months (*p* = 0.04), eight months (*p* = 0.03), and 10 months (*p* < 0.0001) of age in wild-type and *Tuba1a^ND/+^*mice. ***C***, Line graph representing latency to fall on rotarod task at two months (*p* = 0.31), three months (*p* = 0.82), five months (*p* = 0.03), eight months (*p* = 0.004), and 10 months (*p* = 0.04) of age in wild-type and *Tuba1a^ND/+^*mice. Points depict the mean stance width or latency to fall by genotype with SEM. The same mice were analyzed throughout, and a two-way ANOVA was used to assess statistical significance between groups. Number of mice per genotype is as follows: *N* = 2 at two months, *N* = 5 at three months, *N* = 8 at five months, *N* = 12 at eight months, *N* = 8 at 10 months. ***D***, Line graph representing performance over three subsequent rotarod trials in a single testing session at five months of age for *Tuba1a^ND/+^*compared with wild type. Slopes of the lines for wild type and *Tuba1a^ND/+^*were compared by linear regression and were not found to be significantly different (*p* = 0.32, wild-type *R*^2^ = 0.98, *Tuba1a^ND/+^ R*^2^ = 0.89). ***E***, Scatter plot of grip strength assessed in the forelimbs of female five-month-old wild-type and *Tuba1a^ND/+^*mice (*N* = 7, *p* = 0.19). ***F***, Bar graph of total body weight for eight- to 10-month-old *Tuba1a^ND/+^*and wild-type female mice (*N* = 7, *p* = 0.24). ***G***, Bar graph of SUDO scores Bonin et al. (2014) for Von Frey mechanical sensation testing in three- to six-month-old wild-type and *Tuba1a^ND/+^*mice (*N* = 7, *p* = 0.04). ***H***, Bar graph of time to withdraw from thermal stimulus in Hargreaves behavioral analysis on three-month-old wild-type and *Tuba1a^ND/+^*mice (*N* = 7, *p* = 0.19). Sex as a variable did not significantly impact rear stance width or rotarod performance at any time point examined (*N* = 7 females, *N* = 5 males; *p* > 0.05 for all comparisons of male vs female). Sex was also not found to significantly influence sensory behavior (*N* = 7 males, *N* = 2 females; *p* > 0.05 for all comparisons); *p < 0.05, **p < 0.01, ****p < 0.0001. For line graphs, points indicate mean by genotype at each time point ± SEM. For Bar graphs, bars represent mean ± SEM. *p < 0.05; **p < 0.01; ****p < 0.0001.

Having established that neurons from young *Tuba1a^ND^* mutant animals exhibit deficits in microtubule density and organelle trafficking, we next asked whether these lead to impairments at the level of behavior. The *Tuba1a^ND^* mutant allele was identified due to locomotor deficits apparent in homozygous mutant embryos. *Tuba1a^ND^*^/ND^ homozygous substitution induced severe neurodevelopmental abnormalities that impaired motor neuron function and caused perinatal lethality (Gartz Hanson et al., 2016). In contrast, *Tuba1a^ND^*^/+^ embryos survive to adulthood and were indistinguishable from wild-type siblings at the age of weaning (P21). *Tuba1a^ND^*^/+^ embryos exhibit normal presynaptic and postsynaptic motor development within the diaphragm and respond to touch stimulation (Gartz Hanson et al., 2016). However, as *Tuba1a^ND^*^/+^ mice aged, we noticed differences in locomotion that were quantified with gait analysis. While the gait of two-month-old *Tuba1a^ND^*^/+^ mice is not significantly different from wild type (*N* = 2), by three months of age, *Tuba1a^ND^*^/+^ mice develop an ataxic gait, as evidenced by a wider rear stance (Fig. 5*A*,*B*; *N* = 5, *p* = 0.02). *Tuba1a^ND^*^/+^ mice developed impaired motor coordination by five months of age, as assessed by rotarod performance (Fig. 5*C*; *N* = 10, *p* = 0.03). After the initial onset of behavioral phenotypes, *Tuba1a^ND^*^/+^ mice performed worse than wild type at all time points examined (*p* < 0.05 for all). Heterozygous *Tuba1a^ND^*^/+^ mice did not display deficits in motor learning as assessed by the difference in rotarod performance between trials (Fig. 5*D*; *N* = 10, wild-type *R*^2^ = 0.98, *Tuba1a^ND^*^/+^ *R*^2^ = 0.89, *p* = 0.31 by linear regression). Locomotor impairment of these mice impeded more rigorous testing of cognitive function. Importantly, forelimb function, and all other parameters of gait were similar to that measured in wild-type animals (Extended Data Fig. 5-1). Body weights of *Tuba1a^ND^*^/+^ mice were comparable to that of control mice (Fig. 5*F*; *N* = 7, *p* = 0.24), and forelimb muscular strength was not impaired (Fig. 5*E*; *N* = 7, *p* = 0.19). These results indicate that neither altered muscle content nor gross muscle function is responsible for the observed motor deficits, indicating *Tuba1a^ND^*-induced neuronal dysfunction is a likely cause. The hindlimb-specificity of this behavioral phenotype further suggests that neurons with extremely long axons, such as those that innervate the hindlimbs, may be exquisitely sensitive to perturbations of *Tuba1a*.

10.1523/ENEURO.0045-20.2020.f5-1 Extended Data Figure 5-1 *Tuba1a^ND/+^*does not impact forelimb gait and specifically impacts rear stance width. ***A***, Scatter plot of front stance width in five-month-old wild-type and *Tuba1a^ND/+^*mice. ***B***, ***C***, Scatter plot of forelimb (***B***) and hindlimb (***C***) stride length in five-month-old wild-type and *Tuba1a^ND/+^*mice (*N* = 8 mice, *p* > 0.05 for all by *t* test). Download Figure 5-1, TIF file.

*Tuba1a* is expressed in all postmitotic neurons (Paden et al., 1995; Bamji and Miller, 1996), therefore we predicted that behavioral impairments induced by *Tuba1a^ND^*^/+^ substitution would not be restricted to motor neurons. To assess whether *Tuba1a^ND^*^/+^ adult mice had deficits in sensory behavior, we examined hind-paw sensory function using two separate assays in mature mice. Response to hind-paw stimulation with Von Frey filaments revealed an increased threshold for retraction in *Tuba1a^ND^*^/+^ compared with wild-type mice, indicating reduced sensation of mechanical stimuli (Fig. 5*G*; *N* = 7, *p* = 0.04). Additionally, adult *Tuba1a^ND^*^/+^ animals displayed a modest, but not statistically significant decrease in thermal pain sensation of the hind-paws, as measured by a Hargreaves assay (Fig. 5*H*; *N* = 7, *p* = 0.19). These results support our prediction that *Tuba1a^ND^*^/+^-induced behavioral deficits are not restricted to the motor system, but rather appear to preferentially impact the far-reaching neurons that innervate the hindlimbs. The degenerative behavioral phenotypes in *Tuba1a^ND^*^/+^ mice indicate that Tuba1a deficiency has long-lasting consequences for neuronal function.

### TUBA1A deficit impairs hindlimb behavior without apparent neuronal cell death or axon degeneration

**Figure 6.**
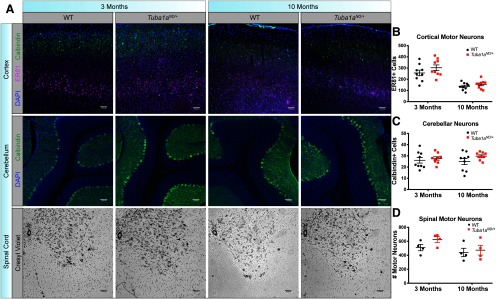
*Tuba1a^ND/+^*does not impact neuronal cell body survival. ***A***, top row, Coronal sections of motor cortex immunolabeled with calbindin (green), ER81 (magenta), and DAPI (blue) in wild-type and *Tuba1a^ND/+^*mice at three months (left) and 10 months (right) of age at 20× magnification. Center row, Sagittal sections of cerebellum labeled with calbindin (green) and DAPI (blue) in wild-type and *Tuba1a^ND/+^*mice at three months (left) and 10 months (right) of age at 20× magnification. Bottom row, Nissl-stained coronal sections of the lumbar spinal cord in wild-type and *Tuba1a^ND/+^*mice at three months (left) and 10 months (right) of age at 4× magnification. ***B***, Scatter plot representing the number of ER81+ Layer V neurons per image in motor cortex at three and 10 months (*N* = 3 mice, *p* = 0.19 and *p* = 0.78, respectively). ***C***, Scatter plot representing the number of calbindin+ Purkinje neurons per image in the cerebellum at three and 10 months (*N* = 3, *p* = 0.94 and *p* = 0.35, respectively). ***D***, Scatter plot representing the number of Nissl+ ventral horn motor neurons per image in the spinal cord at three and 10 months (*N* = 3, *p* = 0.66 and *p* = 0.99, respectively). Motor neurons were identified morphologically. Data points represent ROIs with horizontal line depicting mean with SEM. Data points were nested by animal and analyzed by two-way ANOVA.

**Figure 7.**
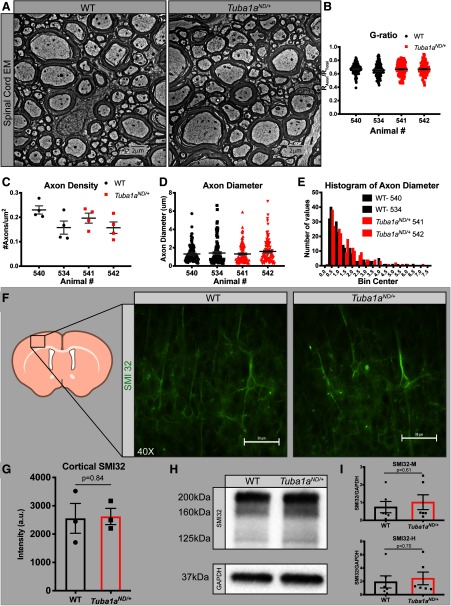
No deficits in axon morphology, myelination, or survival in *Tuba1a^ND/+^*mice. ***A***, Electron micrographs of spinal cord axons in cross-section for adult wild-type (left) and *Tuba1a^ND/+^*(right) mice. Six images per animal and two animals per genotype were assessed for EM analyses. ***B***, Scatter plot displaying myelin thickness measure (G-ratio) for two wild-type and two *Tuba1a^ND/+^*mice (*N* = 2 mice, *n* = 100 axons, *p* = 0.42). ***C***, Scatter plot of axon density quantified in electron micrograph images for adult wild-type and *Tuba1a^ND/+^*mice (*N* = 2 mice, *n* = 6 fields, *p* = 0.72). ***D***, Scatter plot of axon diameter quantified in electron micrograph images for adult wild-type and *Tuba1a^ND/+^*mice (*N* = 2 mice, *n* = 100 axons, *p* = 0.63). ***E***, Histogram representing the distribution of axon diameters from data in ***D***, ***F***, Anti-SMI-32 (non-phosphorylated neurofilament) immunofluorescence images in cortex of adult wild-type (left) and *Tuba1a^ND/+^*mice (right); 40× magnification. Scale bar: 10 μm. ***G***, Bar graph representing SMI-32 intensity in a cortical region of interest for wild-type and *Tuba1a^ND/+^*mice. Data points represent regions of interest, with three animals per genotype assessed (*p* = 0.77). ***H***, Western blotting for SMI-32 in whole-brain lysates from adult wild-type and *Tuba1a^ND/+^*mice. Bands for heavy (200 kDa), medium (160 kDa), and light (125 kDa) chain non-phosphorylated neurofilament shown (top). GAPDH used for normalization (37 kDa; bottom). Data points represent technical replicates with three biological replicates performed. ***I***, Bar graphs representing quantification of SMI-32 medium (right, *p* = 0.61) and heavy (left, *p* = 0.70) expression in whole-brain lysates, normalized to GAPDH. For all graphs, bar or line indicates mean and error bars display SEM.

Complex motor behaviors involve multiple types of motor neurons coordinated through multiple brain regions including the cortex, cerebellum, and spinal cord. We examined survival of neurons that are important for coordinated movement in each of these systems in *Tuba1a^ND^*^/+^ and wild-type mice. Cortical lamination occurs normally in *Tuba1a^ND^*^/+^ mice, and there was no evidence of heterotopic neurons in *Tuba1a^ND^*^/+^ brains (Fig. 6*A*; *N* = 3). To assess cortical motor neuron number, we used the transcription factor ER81 as a marker of Layer V cortical neurons that project to the spinal cord (Yoneshima et al., 2006). We found no difference in the number of cortical motor neurons in Layer V of the motor cortex and no evidence of apoptosis before or after the onset of quantifiable motor deficits in *Tuba1a^ND^*^/+^ and wild type (Fig. 6*A*,*B*; Extended Data Fig. 6-1; *p* = 0.1). We quantified the number of Purkinje neurons, the primary output cells of the cerebellum, and found no difference in Purkinje cell number between genotypes at either time point (Fig. 6*A*,*C*; *N* = 3, *p* = 0.12). Quantification of motor neurons in serial sections through the lumbar spinal cord, which contains neurons that innervate the rear limbs, revealed that there was no alteration to the number of spinal motor neurons in *Tuba1a^ND^*^/+^ animals compared with wild type (Fig. 6*A*,*D*; *N* = 3, *p* = 0.2). In electron micrographs of mature spinal cords from *Tuba1a^ND^*^/+^ and wild-type animals (Fig. 7*A*; *N* = 2 mice), we found no difference in myelin thickness by G-ratio (Fig. 7*B*; *n* = 100 axons, *p* = 0.42), axon density (Fig. 7*C*; *p* = 0.72), or axon diameter (Fig. 7*D*,*E*; *p* = 0.63) at three or 10 months of age. Reduced neuron survival, loss of myelination, and axon degeneration are reported in several movement disorders (De Vos et al., 2008; Murray et al., 2010; Beharry et al., 2014; Brady and Morfini, 2017; Correale et al., 2019; Andreone et al., 2020). However, these results indicate that reduced TUBA1A function does not impact the establishment or maintenance of spinal motor neuron density, axon caliber, or myelination.

10.1523/ENEURO.0045-20.2020.f6-1 Extended Data Figure 6-1 No evidence of apoptosis in *Tuba1a^ND/+^*cortex in young or old mice. ***A***, TUNEL staining (green) with DAPI (blue) in wild-type (left) and *Tuba1a^ND/+^*(center) cortex, with DNase-treated positive control cortex (right). Sections from three-month-old (top) and 10-month-old (bottom) animals are shown. No evidence of increased apoptosis by genotype was detected at either time point. Download Figure 6-1, TIF file.

We examined the possibility that reduced TUBA1A function causes axon degeneration. To evaluate axon integrity in the *Tuba1a^ND^*^/+^ model, we quantified non-phosphorylated neurofilament (SMI32) that is highly abundant in degenerating axons (Watson et al., 1993; Thangavel et al., 2009; Seehusen and Baumgärtner, 2010). We sampled several regions of interest from cortex and observed no increase in SMI32 immunoreactivity in cortical sections from *Tuba1a^ND^*^/+^ mice compared with wild type (Fig. 7*F*,*G*; *N* = 4, *p* = 0.84). We also quantified SMI32 abundance in *Tuba1a^ND^*^/+^ and wild-type whole-brain lysates and detected no difference in SMI32 protein between the two groups (Fig. 7*H*,*I*; *N* = 3, NFT-M *p* = 0.61, NFT-H *p* = 0.70). Overall, these data support the conclusion that reduction of TUBA1A causes adult-onset behavioral deficits without obvious detriment to neuronal survival or morphology.

### NMJ synapses are diminished by prolonged microtubule dysfunction

**Figure 8.**
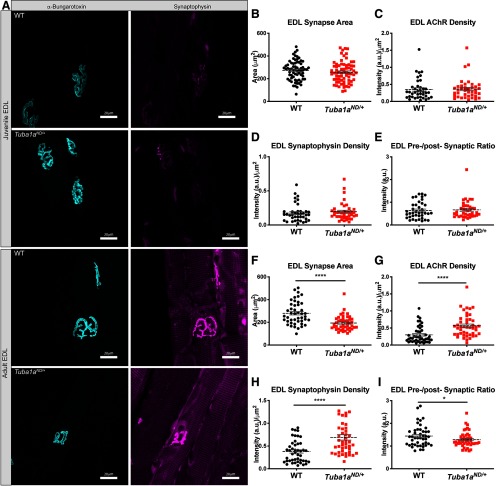
Neuromuscular Junction (NMJ) synapses deteriorate over time in *Tuba1a^ND/+^*mice. ***A***, Teased Extensor digitorum longus (EDL) muscle fibers labeled with α-bungarotoxin (cyan) and synaptophysin (magenta) for one-month-old (top) and one-year-old (bottom) wild-type and *Tuba1a^ND/+^*mice. Muscle fibers from three mice per genotype at each time point were assessed. ***B***, Scatter plot of EDL synaptic (μm^2^) measured from α-bungarotoxin labeling in juvenile wild-type and *Tuba1a^ND/+^*mice (*N* = 3 mice, *n* = 69 synapses, *p* = 0.43). ***C***, Scatter plot of postsynaptic acetylcholine receptors (AChRs) density measured as synaptic α-bungarotoxin intensity in arbitrary units (a.u.) divided by synaptic area in juvenile mice (*N* = 3 mice, *n* = 39 synapses, *p* = 0.89). ***D***, Scatter plot of presynaptic vesicle density measured as synaptic synaptophysin intensity divided by synaptic area in juvenile mice (*N* = 3 mice, *n* = 39 synapses, *p* = 0.54). ***E***, Scatter plot of the ratio of synaptophysin to AChR (presynaptic to postsynaptic) intensity in juvenile wild-type and *Tuba1a^ND/+^*mice (*N* = 3 mice, *n* = 39 synapses, *p* = 0.74). ***F***, Scatter plot of EDL synaptic area measured from α-bungarotoxin labeling in adult wild-type and *Tuba1a^ND/+^*mice (*N* = 3 mice, *n* = 45 synapses, *p* < 0.0001). ***G***, Scatter plot of postsynaptic AChR density measured as synaptic α-bungarotoxin intensity divided by synaptic area in adult mice (*N* = 3 mice, *n* = 45 synapses, *p* < 0.0001). ***H***, Scatter plot of presynaptic vesicle density measured as synaptic synaptophysin intensity divided by synaptic area in adult mice (*N* = 3, *n* = 45, *p* < 0.0001). ***I***, Scatter plot of the ratio of synaptophysin to AChR (presynaptic to postsynaptic) intensity in adult wild-type and *Tuba1a^ND/+^*mice (*N* = 3 mice, *n* = 45 synapses, *p* = 0.03). For all graphs, data points represent individual synapses with line representing mean of data ± SEM. Data were nested by animal and analyzed for statistical significance between genotypes by a two-way ANOVA. *p < 0.05; ****p < 0.0001.

Finally, we examined whether *Tuba1a^ND^*^/+^ impacts synaptic structure at the NMJ. NMJ function is critical to motor output, as it allows for the conversion of electrochemical signaling in neurons to muscle contraction and movement. To assess NMJ synaptic morphology, we labeled acetylcholine receptors (AChRs) with fluorescently conjugated α-bungarotoxin in EDL muscles of both juvenile and adult *Tuba1a^ND^*^/+^ and wild-type hindlimbs. In rodents, NMJ synapses begin forming embryonically, and complete the final stages of synapse maturation, including synaptic pruning and stabilization, between two and three weeks postnatal (Tintignac et al., 2015). Juvenile *Tuba1a^ND^*^/+^ NMJ synapses were evaluated at one month of age, before the onset of quantifiable behavioral deficits, but following the completion of most NMJ development. Wild-type and *Tuba1a^ND/+^* synapses labeled for postsynaptic AChRs as well as presynaptic vesicle marker synaptophysin were evaluated for synaptic area and the relative density of both presynaptic and postsynaptic machinery (Fig. 8*A*). Juvenile *Tuba1a^ND/+^* EDL NMJ synapses were indistinguishable from wild type in synaptic area (Fig. 8*B*; *n* = 69 synapses, *p* = 0.3), density of presynaptic and postsynaptic components (Fig. 8*D*,*C*; *N* = 3 mice, *n* = 39 synapses, *p* > 0.5 for both), and the ratio of presynaptic to postsynaptic machinery (Fig. 8*E*; *n* = 39 synapses, *p* = 0.74). The same analyses were performed for EDL synapses in adult (approximately one year old) wild-type and *Tuba1a^ND/+^*mice. A dramatic reduction in synaptic branching complexity in the *Tuba1a^ND/+^*EDL was immediately apparent, illustrated by the example images (Fig. 8*A*). Further, in adult *Tuba1a^ND/+^* NMJs, synaptic area was found to be significantly reduced compared with wild type (Fig. 8*F*; *N* = 3 mice, *n* = 45 synapses, *p* < 0.0001). Additionally, adult *Tuba1a^ND^*^/+^ synapses contained a higher density of postsynaptic AChRs than wild type (Fig. 8*G*; *p* < 0.0001). The increased AChR density in the adult EDL was also accompanied by an increase in presynaptic synaptophysin (8H), but the ratio of presynaptic to postsynaptic machinery was modestly decreased in *Tuba1a^ND/+^* NMJs compared with wild type, indicating that *Tuba1a^ND/+^* NMJs had lower presynaptic vesicle density relative to the postsynaptic receptor density (Fig. 8; *p* < 0.0001 and *p* = 0.03, respectively). Collectively these data demonstrate that reduced TUBA1A diminishes NMJ size and alters NMJ synapse morphology and functional properties in an age-related manner.

## Discussion

Here, we show that significant reduction in developmental total α-tubulin resulting from a loss of function mutation in *Tuba1a* (*Tuba1a^ND/+^*) causes an adult onset movement disorder. We show that assembly of the microtubule network is altered resulting in disrupted organelle trafficking early in development in cultured *Tuba1a^ND/+^* cortical neurons, suggesting that neuronal microtubule networks are not set up correctly when there is not adequate α-tubulin available (Figs. 1-4). While these aberrant neuronal microtubule networks are sufficient to establish juvenile NMJ synapses similarly to wild type, *Tuba1a^ND/+^* NMJ synapses deteriorate over time (Fig. 8), potentially contributing to adult-onset motor deficits. This work provides the first evidence that the developmental tubulin *TUBA1A* is necessary for adult neuronal function and suggests that mature neurons require *TUBA1A* for proper function throughout their lifetime.

The microtubule cytoskeleton forms the tracks on which motor proteins bind to facilitate intracellular transport. Mitochondria and lysosomes are both transported by dynein motor proteins in the retrograde direction, but differ in the kinesin motors that primarily regulate their anterograde movement, with lysosome transport dominated by kinesin-2 and mitochondrial transport occurring primarily by kinesin-1 (Tanaka et al., 1998; Ligon and Steward, 2000; Hendricks et al., 2010). Thus, examining transport dynamics for these two different organelles allowed us to distinguish between compromised interactions with specific motor proteins or universal effects of a compromised microtubule network. Neither lysosome nor mitochondria velocity was affected by depletion of TUBA1A function, suggesting that motors interact with the microtubule substrate normally (Fig. 4*D*,*H*). Importantly, we did not see differences in amounts of PTMs that are known to affect the activity of motors on their microtubule substrates (Fig. 2*C*,*D*). Thus, this is the first demonstration that reducing available TUBA1A affects organelle trafficking in living neurons.

Mitochondria and lysosomes move with similar average velocities in wild-type neurons, however the dynamics of movement for these two organelle types vary greatly. We observed a larger proportion of stationary mitochondria (60%) than stationary lysosomes (30%) in wild-type neurons (Fig. 4*C*,*G*). The proportion of stationary mitochondria in our cortical cultures was consistent with what has been previously reported in neurons (Ligon and Steward, 2000; Misgeld et al., 2007; Schwarz, 2013). The variation in transport dynamics between these two organelle types could potentially explain the discrepancy in trafficking deficits we observed between lysosomes and mitochondria in *Tuba1a^ND/+^*neurons. Lysosomes are highly motile organelles; therefore, a lysosomal stalling deficit was readily detected in *Tuba1a^ND/+^* neurons (Fig. 4*C*). However, such a deficit was not apparent in the relatively stationary mitochondrial population (Fig. 4*G*). The relatively subtle deficit in pause duration that was observed in mitochondria was not detectable in lysosomes, which had much shorter pause durations on average (Fig. 4*E*,*I*). Despite the discrepancies in how these abnormalities were detected between different organelles, the overall distance traveled by mitochondria and lysosomes was consistently reduced in *Tuba1a^ND/+^* neurons compared with wild type (Fig. 4*F*,*J*). The increase in pausing or stalling behavior (Fig. 4*C*,*I*), combined with the reduced α-tubulin protein during development, and normal neuronal survival (Figs. 2, 6, 7) suggests that reduced availability of TUBA1A is detrimental to adult neuronal function, but not neuronal survival.

The abundance of α-tubulin protein during brain development is reduced in *Tuba1a^ND/+^*brains (Fig. 2). This deficit in α-tubulin reduced the number of growing microtubule (+)-ends per micrometer of neurite in developing *Tuba1a^ND/+^*neurons, indicating the neuronal microtubule network contains a lower density of microtubules in *Tuba1a^ND/+^*mice (Fig. 3). We did not observe differences in neuronal survival, axon caliber, myelination, or markers for axon degeneration in *Tuba1a^ND/+^*compared with wild-type siblings (Figs. 6, 7) suggesting that reduced TUBA1A impacts neuron function rather than neuron survival or axon morphology. Collectively, these results could suggest that adult *Tuba1a* expression may be important for maintenance of microtubule tracks in neurons and mutant *TUBA1A^ND^* microtubules are not sufficient for that requirement. We favor a second model, that the developmental TUBA1A deficit does not allow a neuron to correctly establish microtubule networks of the appropriate density, and these defects cannot be sufficiently repaired to maintain functional NMJ synapses in adulthood despite adequate levels of adult α-tubulin protein (Figs. 2, 3, 8).

Intracellular transport deficits in *Tuba1a^ND/+^*neurons provide a putative mechanism by which reduced TUBA1A impairs neuronal function to cause adult-onset behavioral deficits. However, as cortical neurons are not representative of the adult condition, follow-up studies are needed to determine whether trafficking deficits persist into adulthood in the *Tuba1a^ND/+^*model. Because developmental α-tubulin protein is reduced in *Tuba1a^ND/+^*brains without detectable compensation from any other α-tubulin isotypes (Fig. 2), we propose a model in which early α-tubulin deficits cause formation of inadequate neuronal microtubule tracks that cannot be rescued by subsequent restoration of α-tubulin protein. Over time, the consequences of early tubulin deficiency could become more pronounced and result in impairment of synaptic function and animal behavior. Microtubules in neurons are not singly nucleated from the centrosome, but rather exist in short segments that tile the length of the axons and dendrites (Stiess et al., 2010). When moving cargoes reach the end of a microtubule segment, the motor protein pauses to switch tracks (Yogev et al., 2016). Thus, the observed increase in organelle pausing behavior paired with developmental decreases in α-tubulin protein and microtubule density could indicate that motor proteins in *Tuba1a^ND/+^*neurons encounter microtubule ends more frequently, due to the presence of potential gaps in the tiled microtubule network. Alternatively, while no compensation for loss of TUBA1A was observed at the mRNA level, decreased α-tubulin protein would likely shift the α-tubulin isotype blend at the protein level. It has been previously shown that incorporation of different tubulin isotypes alters microtubule properties, whether directly or through effects on MAP binding (Panda et al., 1994; Sirajuddin et al., 2014; Gadadhar et al., 2017; Honda et al., 2017). Although α-tubulin protein levels are similar to wild type in the adult *Tuba1a^ND/+^*brain, it is unclear whether adult *Tuba1a^ND/+^*microtubule polymers contain normal α-tubulin isotype compositions.

Microtubules and intracellular transport are important for synaptic signaling and maintenance (Kim and Lisman, 2001; Jaworski et al., 2009; Maas et al., 2009; Stephan et al., 2015). Neurons are morphologically complex cells, with processes often extending long distances from the soma, and are thus highly dependent on functional intracellular transport. Transport deficits, such as those observed in the *Tuba1a^ND/+^*model could impair both delivery and clearance of cargoes if persistent over time, leading to neuronal dysfunction. Functional intracellular transport is essential for delivery of mitochondria and synaptic components to the presynapse (Goldstein et al., 2008). Thus, the transport deficits caused by *Tuba1a^ND/+^*substitution could alter synaptic transmission and thereby cause deterioration of synapse morphology, potentially explaining the observed NMJ deficits (Fig. 8). The increased density of both presynaptic and postsynaptic machinery that was observed in the *Tuba1a^ND/+^*NMJ (Fig. 8*G*,*H*) suggests a possible impairment in clearance of vesicles from the presynapse and receptors from the postsynapse. Moreover, the reduction in NMJ synaptic area (Fig. 8*F*) and branching complexity indicates that the increased density of AChRs and presynaptic vesicles may be a form of compensation for improperly functioning synapses. This hypothesis is further supported by the observed dysregulation of presynaptic to postsynaptic component ratios in adult *Tuba1a^ND/+^*synapses (Fig. 8*I*). NMJ synapses are generally quite stable over time, but are known to undergo morphologic changes in response to processes that alter synaptic transmission, such as diseases of the neuromuscular system and aging (Murray et al., 2008, 2010; Jones et al., 2017; Liu et al., 2017). Decreased NMJ size explains impaired motor behaviors in *Tuba1a^ND/+^* mice (Fig. 5*A–C*). However, given that *Tuba1a* is expressed in all neurons, and *Tuba1a^ND/+^*mice also exhibited degenerative sensory deficits (Fig. 5*G*,*H*), we do not expect that the NMJ is the only synapse type affected by deficiency in TUBA1A, but this idea remains to be tested. The prominence of hindlimb-specific behavioral phenotypes in the *Tuba1a^ND/+^*mouse model suggests that axon length may predict sensitivity to tubulin deficiency.

Microtubule dysfunction, including intracellular trafficking defects, have been described in nearly every neurodegenerative disease of the nervous system, and can precede other signs of neuronal dysfunction (Reynolds et al., 2004; De Vos et al., 2008; Cartelli et al., 2010; Matamoros and Baas, 2016; Brady and Morfini, 2017). Although there is abundant evidence of microtubule dysfunction in neurodegenerative disease, it has been difficult to pinpoint how or whether the microtubule cytoskeleton contributes to disease etiology. Microtubule dynamics, PTMs, and MAP interactions become dysregulated in neurodegenerative diseases such as Alzheimer’s and Parkinson’s disease, and amyotrophic lateral sclerosis (ALS); however, it has remained unclear as to whether cytoskeletal dysfunction is causative of disease or merely correlated with its progression (Cartelli et al., 2010; Rogowski et al., 2010; Brunden et al., 2011; Dubey et al., 2015; Matamoros and Baas, 2016). Mutations that disrupt the neuronal α-tubulin TUBA4A have been identified familial and sporadic ALS (Rademakers and van Blitterswijk, 2014; Smith et al., 2014; Pensato et al., 2015; Li et al., 2018). The mechanisms by which TUBA4A mutations may cause neurodegeneration remain unclear. Using the *Tuba1a^ND/+^*model, we have demonstrated that loss-of-function in a major neuronal α-tubulin is sufficient to induce adult-onset behavioral degeneration. Further, we have shown that *Tuba1a^ND/+^*does not induce neuronal death or degeneration, but rather causes synaptic abnormalities, potentially by impairing intracellular transport. We provide direct evidence that deficits in microtubule function can cause degenerative neuronal pathology at the molecular, cellular, and behavioral level.

## Synthesis

Reviewing Editor: Deanna Smith, University of South Carolina Decisions are customarily a result of the Reviewing Editor and the peer reviewers coming together and discussing their recommendations until a consensus is reached. When revisions are invited, a fact-based synthesis statement explaining their decision and outlining what is needed to prepare a revision will be listed below. The following reviewer(s) agreed to reveal their identity: Laura Volpicelli-Daley, Dianna Willis. Note: If this manuscript was transferred from JNeurosci and a decision was made to accept the manuscript without peer review, a brief statement to this effect will instead be what is listed below.

Dear Dr. Bates,

I am pleased to inform you that your manuscript entitled “Reduced TUBA1A tubulin causes defects in trafficking and impaired adult motor behavior” has been deemed acceptable for publication in eNeuro. The reviewers indicated that the manuscript advances the field by demonstrating an important role for Tub1A in adult neurons, and advances our understanding of how microtubule assembly during development can impact adult neuronal function. There was the suggestion that adult DRG neurons might be a better system for this study, but that the rationale for use of embryonic neurons was adequately addressed. There is one issue that you should fix regarding a figure legend. The legend in Figure C3 states: “Representative kymograph plots generated from GFP-MACF43 images in wild type (top) and Tuba1aND/+ (bottom) neurons", should be changes to “Representative kymograph plots generated from GFP-MACF43 images in wild type (left) and Tuba1aND/+ (right) neurons”. Please check on this.
