# Supplementary figures and images for: Reduced TUBA1A Tubulin Causes Defects in Trafficking and Impaired Adult Motor Behavior
Source: eNeuro. 2020 Apr 27;7(2):ENEURO.0045-20.2020. doi: 10.1523/ENEURO.0045-20.2020 (PMC7218002; doi:10.1523/ENEURO.0045-20.2020)

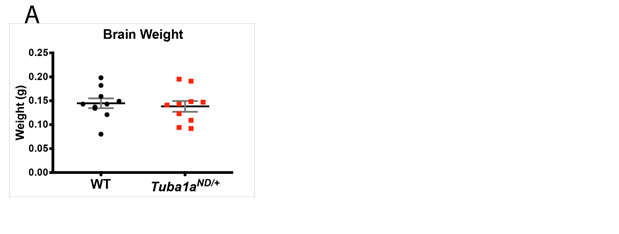

Supplement: Extended Data Figure 2-1 — A, Tuba1aND/+does not alter brain weight at birth. Scatter plot of brain weight for Tuba1aND/+and wild-type mice at P0–P2 (N = 10 mice, p = 0.68 by t test). Weights were recorded from frozen, dissected brains. Download Figure 2-1, TIF file. [file enu-eN-NWR-0045-20-s01.tif]

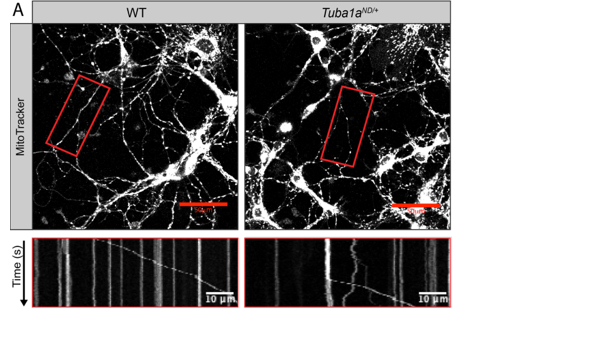

Supplement: Extended Data Figure 4-1 — Tuba1aND/+impairs mitochondrial transport by increasing pause duration. A, Still images from time-lapse microscopy of DIV3 cortical neurons labeled with MitoTracker dye to mark mitochondria in wild-type and Tuba1aND/+ neurons. B, Insets show representative kymograph plots of mitochondrial movement over time within select neurites for wild type (top) and Tuba1aND/+(bottom). Download Figure 4-1, TIF file. [file enu-eN-NWR-0045-20-s03.tif]

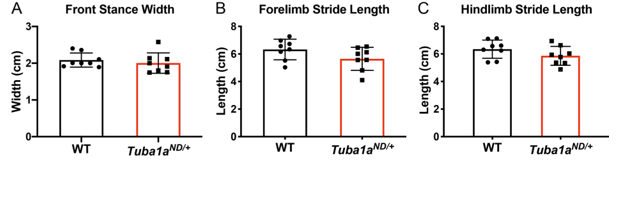

Supplement: Extended Data Figure 5-1 — Tuba1aND/+does not impact forelimb gait and specifically impacts rear stance width. A, Scatter plot of front stance width in five-month-old wild-type and Tuba1aND/+mice. B, C, Scatter plot of forelimb (B) and hindlimb (C) stride length in five-month-old wild-type and Tuba1aND/+mice (N = 8 mice, p > 0.05 for all by t test). Download Figure 5-1, TIF file. [file enu-eN-NWR-0045-20-s02.tif]

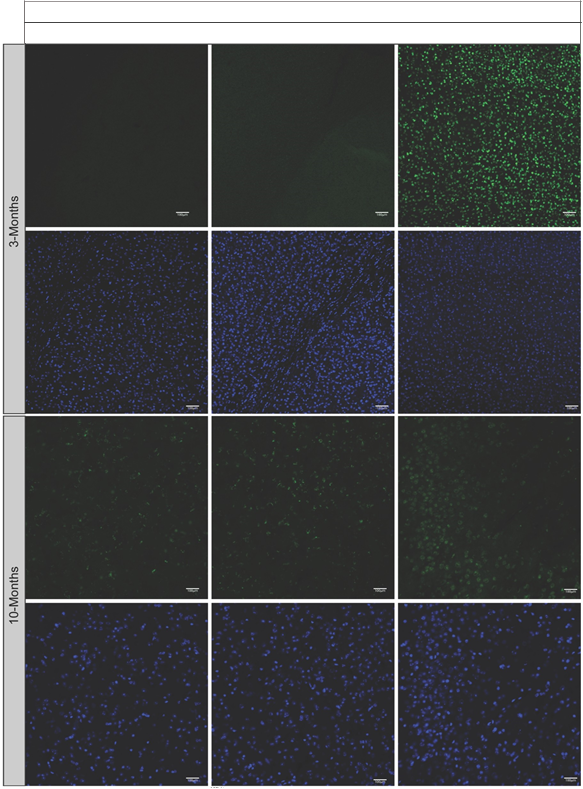

Supplement: Extended Data Figure 6-1 — No evidence of apoptosis in Tuba1aND/+cortex in young or old mice. A, TUNEL staining (green) with DAPI (blue) in wild-type (left) and Tuba1aND/+(center) cortex, with DNase-treated positive control cortex (right). Sections from three-month-old (top) and 10-month-old (bottom) animals are shown. No evidence of increased apoptosis by genotype was detected at either time point. Download Figure 6-1, TIF file. [file enu-eN-NWR-0045-20-s04.tif]
